# Characterizing structural features of cuticle-degrading proteases from fungi by molecular modeling

**DOI:** 10.1186/1472-6807-7-33

**Published:** 2007-05-18

**Authors:** Shu-Qun Liu, Zhao-Hui Meng, Jin-Kui Yang, Yun-Xin Fu, Ke-Qin Zhang

**Affiliations:** 1 Laboratory for Conservation and Utilization of Bio-resources, Yunnan University, Tel. 86-871-5034878, Fax. 86-871-5034838, Yunnan, Kunming 650091, PR China; 2 Department of Cardiology, No. 1 Affiliated Hospital, Kunming Medical College, Yunnan, Kunming, 650032, PR China; 3 Human Genetics Center, School of Public Health, The University of Texas Health Science Center, 1200 Herman Pressler, Room E453, Houston, Texas 77030, USA

## Abstract

**Background:**

Serine proteases secreted by nematode and insect pathogenic fungi are bio-control agents which have commercial potential for developing into effective bio-pesticides. A thorough understanding of the structural and functional features of these proteases would significantly assist with targeting the design of efficient bio-control agents.

**Results:**

Structural models of serine proteases PR1 from entomophagous fungus, Ver112 and VCP1 from nematophagous fungi, have been modeled using the homology modeling technique based on the crystal coordinate of the proteinase K. In combination with multiple sequence alignment, these models suggest one similar calcium-binding site and two common disulfide bridges in the three cuticle-degrading enzymes. In addition, the predicted models of the three cuticle-degrading enzymes present an essentially identical backbone topology and similar geometric properties with the exception of a limited number of sites exhibiting relatively large local conformational differences only in some surface loops and the N-, C termini. However, they differ from each other in the electrostatic surface potential, in hydrophobicity and size of the S4 substrate-binding pocket, and in the number and distribution of hydrogen bonds and salt bridges within regions that are part of or in close proximity to the S2-loop.

**Conclusion:**

These differences likely lead to variations in substrate specificity and catalytic efficiency among the three enzymes. Amino acid polymorphisms in cuticle-degrading enzymes were discussed with respect to functional effects and host preference. It is hoped that these structural models would provide a further basis for exploitation of these serine proteases from pathogenic fungi as effective bio-control agents.

## Background

Proteases are enzymes that catalyze the hydrolysis of peptide bonds in other proteins and are often used in biotechnology, industry, and agriculture as bio-control agents against parasites. Serine proteases (EC 3.4.21.-) are present in virtually all organisms and exist as two major families, the trypsin-like (EC 3.4.21.4) and the subtilisin-like (EC 3.4.21.14) families. These two families have independently evolved with a similar catalytic mechanism which has been widely investigated [1-4]. Although the overall fold of various serine proteases may differ, they all follow the same mechanism of action through an identical stereochemistry of the catalytic triad and oxyanion hole. In this mechanism, the Ser functions as the primary nucleophile and the His plays a dual role as the proton donor and acceptor at different steps in the reaction. The role of Asp is believed to bring the His into the correct orientation to facilitate the nucleophilic attack by the Ser. The role of the oxyanion hole is to stabilize the developing negative charge on the oxygen atom of the substrate during the formation of the tetrahedral intermediate [5-7].

Serine proteases secreted by nematophagous and entomophagous fungi are one of the important virulence determinants in the infection processes of invertebrate mycopathogens [8,9]. They function mainly to degrade insect and nematode cuticles. These cuticles comprise primarily chitin fibrils embedded in a protein matrix, in which the quantity and type of proteins vary between species, tissues and growth stages of insects and nematodes [10,11]. During the infection process, an initial mechanical pressure may play a role but of major importance is the subsequent enzymatic degradation of cuticles within the immediate vicinity of the infection hyphae. Being accompanied by the enzymatic degradation, the cuticles are penetrated and the prey is invaded and eventually digested by fungi. Associated with the ability to penetrate the complex and variable cuticle structures, the pathogenic fungi produce a number of different cuticle-degrading enzymes such as lipases, chitinases and at least four different classes of proteases [12,13]. Among these proteases the most extensively studied insect cuticle-degrading protease is a serine protease, PR1, produced by an entomopathogenic fungus *Metarhizium anisopliae *[14,15]. PR1 is a crucial factor in the infection of the insect because it is produced more rapidly and is in a higher concentration than the other cuticle-degrading enzymes [16]. Furthermore, it has been observed, using gold-labeled antibodies, that the PR1 is secreted by the *Metarhizium anisopliae *during penetration of the host cuticle [17]. A similar protease, VCP1 from the nematophagous fungus, *Pochonia chlamydosporia *(Syn. *Verticillium chlamydosporium*), has also been observed to play an important role during the infection process of nematode eggs [18,19]. More recently, an alkaline serine protease, Ver112 from the nematophagous fungus *Lecanicillium psalliotae *exhibiting cuticle-degrading and nematicidal activity was cloned and sequenced by our laboratory [9,20].

Cloning and sequencing of genes encoding the three proteases (PR1, VCP1 and Ver112) have shown that they have a high degree of sequence similarity with each other and belong to the proteinase K family of subtilisin-like serine proteases (subtilases) which is a large family of endopeptidases found only in fungi and Gram-negative bacteria [3]. These enzymes show conservation of the Asp-His-Ser catalytic triad and catalytic domain. However, the three-dimensional (3D) structures have not been resolved for any of the cuticle-degrading proteases so far by either X-ray crystallography or nuclear magnetic resonance (NMR) techniques. A thorough understanding of the 3D structures of these enzymes can provide greater insight into the mechanism and specificity of enzyme catalysis which would guide future targeted mutagenesis for improved efficiencies.

In this paper, the 3D structural models of the mature enzymes for PR1, VCP1, and Ver112 were modeled based on the available high resolution crystal structure (at 0.98 Å) of Proteinase K using the homology modeling technique. Based on both the multiple sequence alignment and structural models, predictions were made for these cuticle-degrading enzymes concerning calcium-binding site, disulfide bridge, salt bridge, hydrogen bond, aromatic interaction, substrate-binding site and electrostatic surface potential. The physiochemical and geometrical properties of the three cuticle-degrading enzymes and the template protease K were compared to identity factors that might contribute to substrate specificity and host preference by these fungi.

## Results

### Comparison of physiochemical properties

The calculated physiochemical properties and the experimentally determined optimal reaction conditions of the three cuticle-degrading enzymes and proteinase K are listed in Table 1. Cuticle-degrading enzymes share certain physiochemical properties such as comparable amino acid residue number (~280), low molecular weight (~28.6 kDa), high pI value (pI > 7.7), comparable EXTCOEF value, and susceptivity to inhibition by the phenyl methane sulfonyl fluoride (PMSF). Enzyme kinetics assays with substrates such as Suc-Ala-Ala-Ala-p-nitroaniline, Suc-Ala-Ala-Pro-Phe-p-nitroaniline and casein have revealed that they are all alkaline extracellular serine proteases with an optimum activity at pH 8–10 and are highly active at temperatures around 60°C [9,14,18]. The amino acid sequence identities of PR1, VCP1 and Ver112 with respect to 1IC6 are 66.4%, 62.5%, 64.1%, respectively. These similar properties are consistent with earlier propositions that these cuticle-degrading proteases from different fungi are members of the proteinase K family of subtilisin-like serine proteases.

### Model description and structural comparison

**Figure 1:**
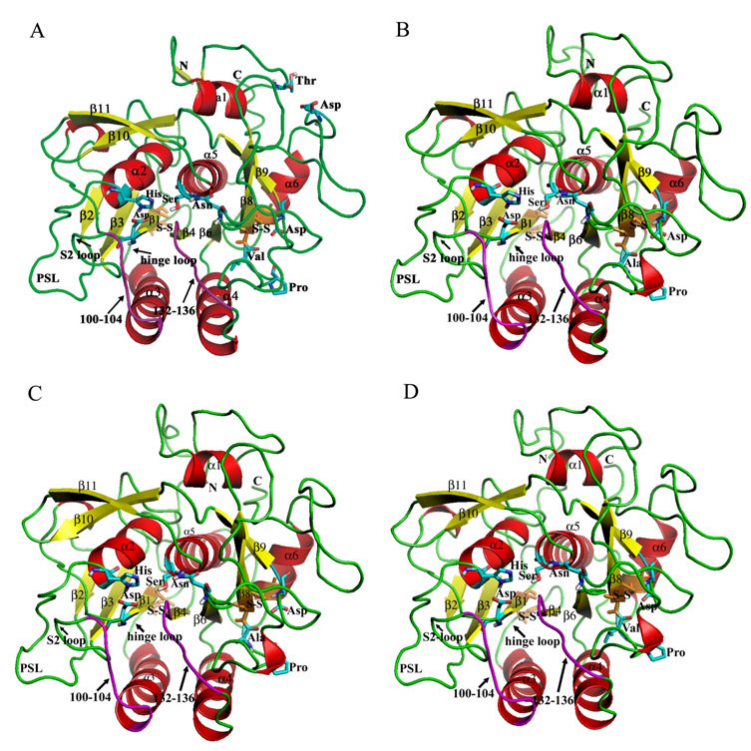
**Ribbon diagrams of the proteinase K and the homology models of cuticle-degrading proteases: (A) 1IC6, (B) PR1, (C) VCP1 and (D) Ver112**. The α helices are colored red, β strands are colored yellow, loops are colored green, substrate-binding segments 100–104 and 132–136 (1IC6 numbering) are colored purple, and disulfide bridges are colored orange. The residues of catalytic triad (Asp39, His69 and Ser224), oxyanion hole (Asn161), disulfide bridges (S-S), and calcium binding sites are represented as stick models. The Ca1 site comprising Pro175, Val/Ala177 and Asp200 is present in all four proteases while the Ca2 site comprising Thr16 and Asp260 is present only in 1IC6.

**Figure 2:**
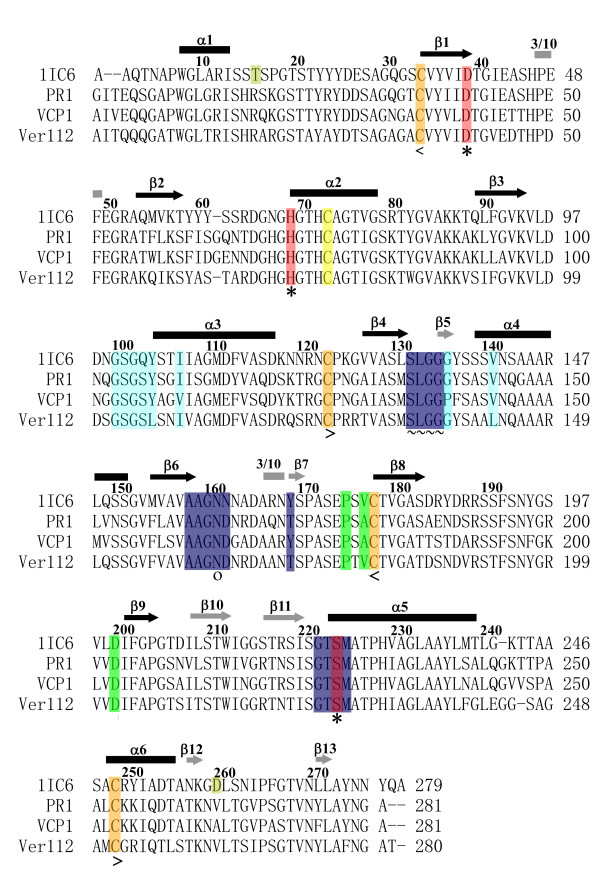
**Multiple sequence alignment of the 1IC6, PR1, VCP1 and Ver112**. The common secondary structure elements are marked with black arrows for parallel β strands, gray arrows for antiparallel β strands, black rods for α helices, and gray rods for 3/10 helices. Residues belonging to the S1 site are shaded blue and residues belonging to the S4 site are shaded light blue. ~ represents residues that are involved in both the S1 and S4 sites. Residues forming the catalytic triad (Asp39, His69 and Ser224, 1IC6 numbering) are shaded red and marked with an *. The oxyanion hole residue Asn161 is marked with o. Cysteines involved in the disulfide bridge are shaded orange and marked with < and > (<Cys34–Cys123> and <Cys178–Cys249>), the free Cys73 is shaded yellow. Residues forming the "strong" calcium binding site (Pro175, Val/Ala177 and Asp200) Ca1 are shaded green and residues forming the weak calcium binding site Ca2 (Thr16 and Asp260) which are unique for 1IC6 are shaded pale green.

Figure 1 shows the 3D structure/models of 1IC6, PR1, VCP1 and Ver112. The structural organizations of these three fungal proteases are almost identical to that of 1IC6. Briefly, these models show the α/β scaffold characteristics of subtilisin-like serine proteases, consisting of six α helices, two 3/10 helices, a seven-stranded parallel β sheet, and three two-stranded antiparallel β sheets. The common secondary structure elements of the four proteases, determined using the program DSSP, are shown in the multiple sequence alignment (Figure 2). The 1IC6 contains five cysteines, out of which two disulfide bridges are formed by residues Cys34–Cys123 and Cys178–Cys249, whereas the Cys73 is free. All three cuticle-degrading enzymes also contain five cysteines that occupy the same positions as the 1IC6 within the multiple sequence alignment (Figure 2). Modeling of the 3D structures reveals the presence of the two disulfide bridges in the three cuticle-degrading enzymes (Figure 1). As in proteinase K, the free SH group of Cys73 in models of the three investigated proteases is located in the region close to the active site and just below the plane of the functionally important His69 imidazole. In addition, the free sulfur atom of Cys73 is engaged in several close contacts with active site residues (His69(O), Ser224(O), and Ser132(O_γ_)). However, the role of the Cys73 is not yet understood. The active site of 1IC6 consists of the catalytic triad Asp39, His69 and Ser224 as well as the oxyanion hole residue Asn161, which are absolutely conserved among the cuticle-degrading enzymes (Figure 1 and 2). The substrate-recognition region in 1IC6 are mainly formed by two polypeptide segments, 100–104 and 132–136, respectively, where the P4-P1 segment of the substrate is accommodated as the central strand to form a three-stranded antiparallel β sheet with residues in the S4-S1 sites of the enzyme. The substrate-recognition/binding sites are well conserved among the four enzymes with subtle amino acid changes in the S1 and S4 pockets (see Figure 2, for details, "Substrate-binding region and electrostatic surface potential").

Pairwise least square superpositions of the four structural models give backbone root mean deviation (RMSD) values of 0.04–0.75 Å. The structural resemblance with regard to the RMSD value is in the following order: PR1-VCP1(0.04 Å) > Ver112-PR1 (0.34 Å) > VCP1-Ver112 (0.35 Å) > Ver112-1IC6 (0.58 Å) > 1IC6-PR1 (0.75 Å) = 1IC6-VCP1 (0.75 Å), indicating that the models of the cuticle-degrading enzymes are more similar to each other than to 1IC6. This roughly reflects the amino acid sequence identities between them. The sequence identities of PR1-VCP1, Ver112-PR1, VCP1-Ver112, Ver112-1IC6, 1IC6-PR1, and 1IC6-VCP1 are 78.6%, 69.5%, 64.3%, 64.1%, 66.4%, and 62.5%, respectively.

**Figure 3:**
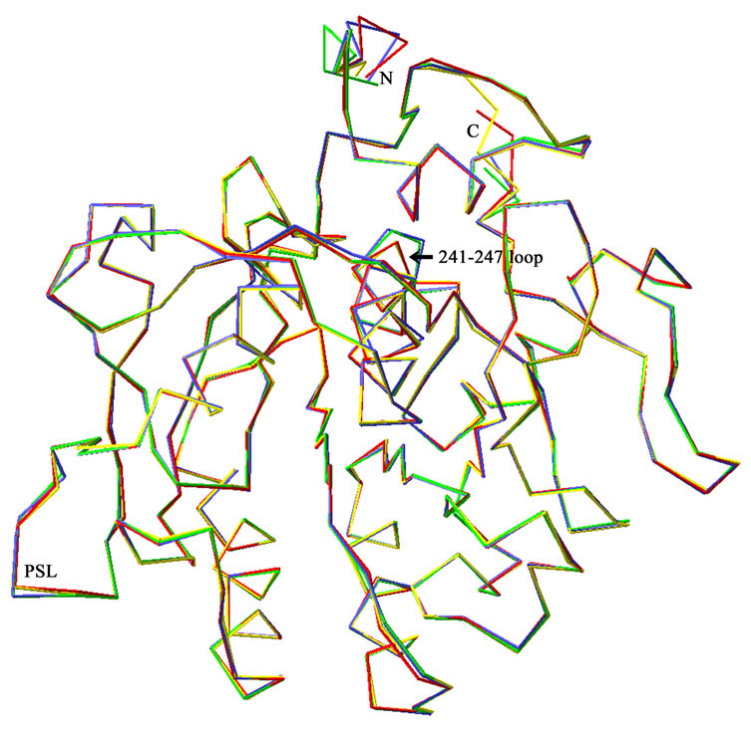
**Backbone superimposition of the 1IC6 (yellow), PR1 (blue), VCP1 (green) and Ver112 (red)**. The four regions (N-terminus, C-Terminus, PSL and 241–247 loop) exhibiting relatively large local conformational differences in the backbone fold are labeled.

Figure 3 shows the superimposed models of the four proteases, in which four regions can be observed to exhibit relatively large local conformational differences in the backbone fold. The most distinct differences can be found in the N- and C-terminal regions, probably because of amino acid insertions in the N-termini and residue deletions in the C-termini relative to the template (Figure 2). The other two regions exhibiting different conformation also involve insertions or deletions in the loop regions (Figure 2 and 3). For example, both PR1 and VCP1 have an insertion of one residue in the polar surface loop (PSL) comprising residues 59–68. PR1, VCP1 and Ver112 have one residue insertion in the surface loop comprising residues 241–247, and Ver112 has one residue deletion at position 243. The few insertions or deletions occurring only in the loop regions, together with the high degree of sequence similarities, might explain why the three proteases present virtually identical obtained structural models. In addition, an analysis of the geometrical properties reveals that the four structure/models have parallel SASA, Rg, NHB, NNC, NSB, SSE and potential energy (Table 2).

### Prediction of Ca^2+ ^binding sites

**Figure 4:**
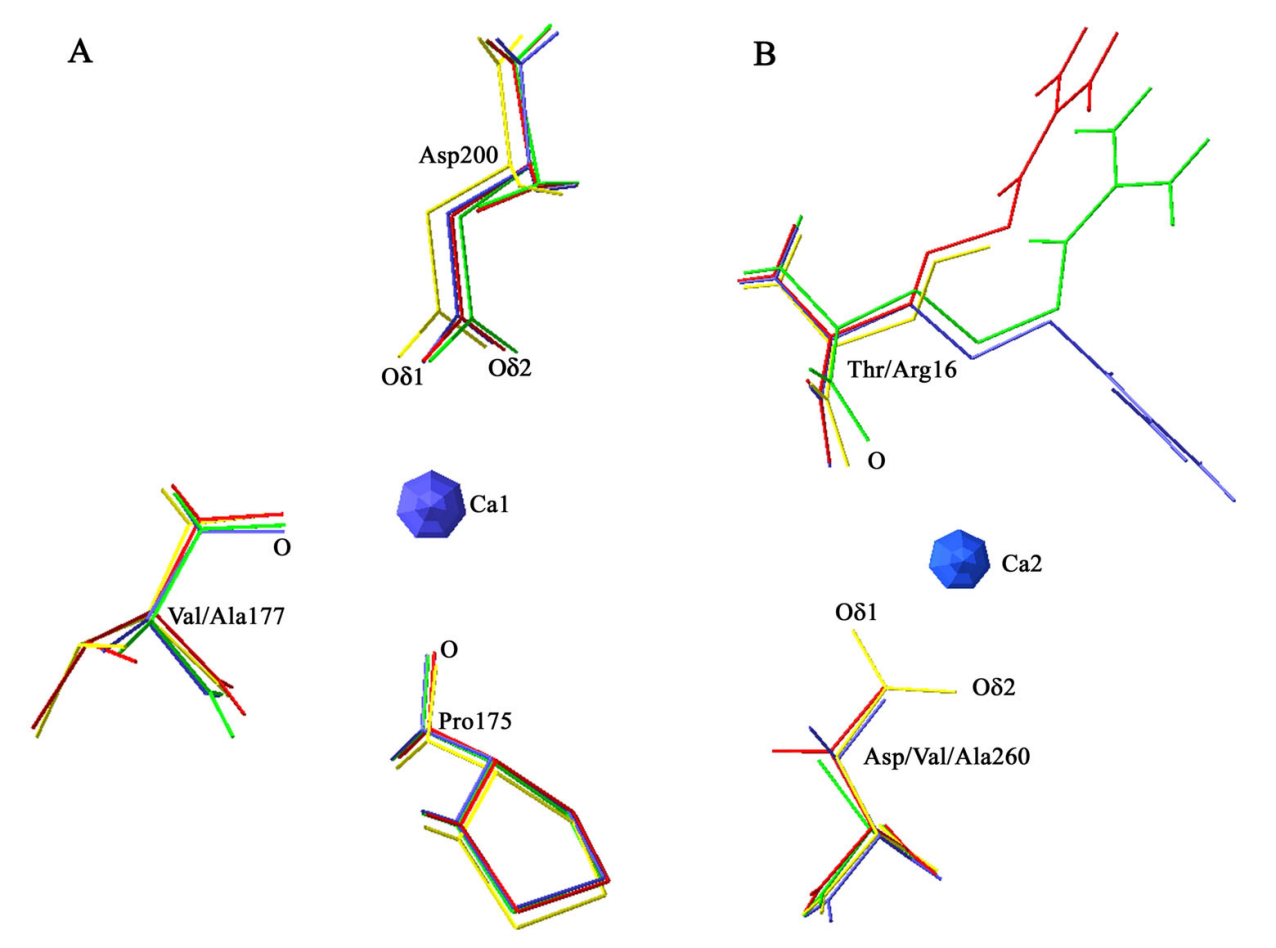
**Superimposition of the calcium binding sites of 1IC6, PR1, VCP1 and Ver112**. (A) Ca1 site. The calcium ion is coordinated by the carboxyl groups of Asp200 and the carbonyl oxygen atoms of Pro175 and Val/Ala177 (1IC6 numbering) in all four proteases, with residues from 1IC6, PR1, VCP1 and Ver112 colored yellow, blue, green and red, respectively. (B) Ca2 site. The calcium ion is coordinated by the carbonyl oxygen atom of Thr16 and carboxyl groups of Asp260 only in 1IC6 (yellow), with the Arg16 and Val/Ala260 in PR1 (blue), VCP1 (green) and Ver112 (red) also displayed to highlight their differences with the Ca2 of 1IC6.

The presence of Ca^2+ ^binding sites is a feature shared by members of the subtilisin-like family. It has been observed that Ca^2+ ^binding enhances the thermal stability of subtilisins which in turn increases their resistance to proteolysis [21,22]. There are two Ca^2+ ^binding sites in the high resolution crystal structure of 1IC6 [23]. The so-called strong binding site Ca1 is formed by the O_δ1 _and O_δ2 _of Asp200 and the carbonyl oxygen atoms of Pro175 and Val177. This strong Ca1 site is involved in stabilizing and bridging loops between β7-β8 and β8-β9. Ca2 is a weak binding site formed by the O_δ1 _and O_δ1 _of Asp260 and the carbonyl oxygen atom of Thr16. This binding stabilizes the N- and C-terminal regions of the molecule. With regard to the Ca1 site, residues at positions 175 and 200 are conserved among the four enzymes. The only exception is that Val177 in 1IC6 is replaced by Ala in PR1 and VCP1 (Figure 2). However, the carbonyl group of Val177 coordinates the Ca^2+ ^and the replacement of Val177 by Ala should have a minor effect on calcium binding. It is likely, therefore, that this strong Ca1 site should also be found in the structures of PR1 and VCP1 (as well as Ver112). Figure 4A shows the superimposed Ca1 sites of the four enzymes. With regard to the Ca2 site, the Thr16 in 1IC6 is replaced by Arg in the three cuticle-degrading enzymes and the Asp260 in 1IC6 is replaced by Val in PR1 and Ver112 and by Ala in VCP1. Thus, the weak Ca2 site is not likely present in the cuticle-degrading enzymes because Val/Ala260 contains no carboxyl side chain (Figure 4B).

### Disulfide bridges

Multiple sequence alignment and structural models suggest that there are two disulfide bridges occupying the same positions in each of the four proteases. One of the disulfide bridges, Cys34–Cys123, connects β1 carrying the catalytic residue Asp39 and a loop connecting α3 and β4. Although this loop is far away from the catalytic triad residues and substrate-binding regions, it links α3 located next to the substrate-binding segment 100–104 and β4 preceding the substrate-binding segment 132–136. As a result, this loop may function as a "hinge" responsible for modulating the orientation of the substrate-binding segments. Thus the Cys34–Cys123 can be thought to get involved in stabilizing the regions close to the catalytic residue Asp39 and substrate-binding sites. In addition, an absolutely conserved salt bridge, Asp117:Arg121, is also found to participate in stabilizing the "hinge" loop. The other disulfide bridge, Cys178–Cys249, connecting β8 located next to the S1 pocket and α6 situated close to the C-terminus, makes the peripheral α6 anchor to the rest of the molecule and contributes to the stability of the C-terminal region. In addition, Cys178–Cys249 may also influence the orientation of the S1 residues via a loop comprising residues 171–177.

### Hydrogen bonds and salt bridges

The numbers of hydrogen bonds/salt bridges in 1IC6, PR1, VCP1 and Ver112 are 223/14, 189/12, 189/12, and 195/13, respectively (Table 2). Hydrogen bonds play an important role in the overall stability of the protein due to their large number and wide distribution. In contrast, the contribution of salt bridges to protein stability is more localized [24]. In this paper, we mainly focus on the salt bridges that contribute to the stability of local structures, and on the number and type of hydrogen bonds and salt bridges that are close to or within the substrate-binding sites.

A shared salt bridge network Arg12:Asp187:Lys/Arg18 is found in the three cuticle-degrading enzymes to participate in stabilizing the N-terminal region which is stabilized by Ca2 in 1IC6. Another common salt bridge network, Arg52:Glu50:Lyr/Arg80, is found in all four enzymes, connecting and stabilizing the surface loops between a 3/10 helix (residues 47–49) and β2 and between α2 and β3. As described above, the absolutely conserved salt bridge, Asp117:Arg121, together with the disulfide bridge Cys34–Cys123, anchors and stabilizes the "hinge" loop. In 1IC6, PR1 and VCP1, there are two successive negatively charged residues, Asp26 and Glu/Asp27, in a loop connecting α1 and β1, and these two residues can form at least two salt bridges with two successive positively charged residues, Lys86 and Lys87 in a loop connecting α2 and β3. In contrast, Ver112 contains just one salt bridge at the equivalent positions due to the replacement of the negatively charged residue at position 27 with a neutral residue, Thr. In addition, a salt bridge Asp112:Arg147 is found in both 1IC6 and Ver112 to bridge α3 and α4, which are directly connected to the substrate-binding segments comprising residues 100–104 and 132–136. Although the negatively charged residues (Asp/Glu112) are conserved among the four proteases, the replacement of Arg147 with Ala in both PR1 and VCP1 disrupts the formation of this salt bridge. In PR1 and VCP1, a salt bridge network, Lys250:Asp254:Lys251, is found to participates in stabilizing the C-terminal helix α6. However, only one salt bridge, Arg250:Asp254, is found in α6 of 1IC6, and no salt bridge is found in α6 of Ver112. Interestingly, a common disulfide bond Cys178–Cys249 is found to participate in stabilizing the α6 in all four investigated protease models.

There are at least one positively charged residue, Arg, and one negatively charged residue, Asp, in a region comprising residues 162–169 which is part of, or in close vicinity to the S1 pocket. In this region, 1IC6 contains a salt bridge, Asp165:Arg167, PR1 and Ver112 share a salt bridge, Asp162:Arg164, and VCP1 contains a salt bridge network comprising three residue, Asp162:Arg168:Asp165. The number of hydrogen bonds in this region is 8, 6, 5, and 4 in 1IC6, PR1, Ver112, and VCP1, respectively. These salt bridges and hydrogen bonds contribute to the structural stability of this region and may cause the P1 residue of the substrate to adopt the correct orientation to facilitate nucleophilic attack by the Ser. Interestingly, the observed reduction in the hydrogen bond number within this region for VCP1 likely is compensated by the salt bridge networks involving the three residues, Asp162:Arg168:Asp165.

The charged residue clusters are found in a loop comprising residues 59–68 and in a region comprising residues 94–98. The loop 59–68 preceding the catalytic residue His69 contains abundant polar residues and resides on the molecular surface, hereafter it is referred to as the polar surface loop (PSL). The loop 95–101 is part of, or in close proximity to the S2 site, and hereafter it is referred to as the S2-loop. In both PR1 and VCP1, there is one salt bridge network, Asp65:Lyr94:Asp97, that bridges the PSL and the S2-loop together and hence stabilizes them. In 1IC6 and Ver112, three salt bridge networks, Asp65:Lyr94:Asp97, Asp65:Lyr94:Asp98 and Arg64:Asp98:Lyr94 are responsible for stabilizing these two loops. Interestingly, an absolutely conserved hydrogen bond network Asp97-[Gly100, S101] which participates in stabilizing the S2-loop is found in all the four enzymes. In addition to Asp65:Lyr94 and Arg64:Asp98, another salt bridge involved in stabilizing the PSL in 1IC6 and Ver112 is Glu43:Arg64. This salt bridge is not found in the PR1 and VCP1 due to the replacement of the Arg64 by a neutral residue, Asn or Thr. Furthermore, there is an absolutely conserved hydrogen network Asp65-[Gly68, Thr71] involved in stabilizing the PSL in the four enzymes, and the number of hydrogen bonds participating in stabilizing the PSL is 10, 9, 7 and 8 in 1IC6, PR1, VCP1 and Ver112, respectively. Taken together, it is thought that the abundant hydrogen bonds in combination with a small quantity of salt bridges provide determinants for the stabilization of the PSL. It has also been observed that the PSL can be stabilized by calcium in two proteinase K-like enzymes, the VPRK from *Vibrio *sp. PA44 [PDB:1SH7] [24] and the thermitase from *Thermoactinomyces vulgaris *[PDB:1THM] [25].

### Aromatic residues and interactions

Clustered aromatic residues with potential interactions between them assist in enhancing the thermal stability of the protein [4]. Aromatic residues that are conserved among the four enzymes are at positions 8(Trp), 23(Tyr), 25(Tyr), 36(Tyr), 49(Phe), 59(Tyr/Phe), 82(Tyr/Trp), 113(Phe/Tyr), 137(Tyr/Phe), 192(Phe), 195(Tyr/Phe), 202(Phe), 212(Trp), 236(Tyr), 274(Tyr/Phe), and 277(Tyr/Phe). Out of them two absolutely conserved aromatic interactions, Tyr23–Tyr25 and Tyr/Phe59-Phe/Tyr113, are found in all four protease models. The aromatic ring stack of Tyr23–Tyr25 can contribute to the stabilization of the N-terminal region, and Tyr/Phe59-Phe/Tyr113 can contribute to the stabilizations of the PSL and α3. The α3 is in close proximity to the substrate-binding segment 100–104. A shared aromatic interaction, Tyr36-Phe/Tyr91, observed in 1IC6, PR1 and Ver112 can potentially contribute to the stabilizations of both β1 and β3. However, this aromatic interaction does not occur in VCP1 due to the replacement of the aromatic residue Phe or Tyr at position 91 by Leu in VCP1. Another aromatic interaction Tyr60-Tyr61 involved in stabilizing PSL is found only in 1IC6.

### Substrate-binding region and electrostatic surface potential

**Figure 5:**
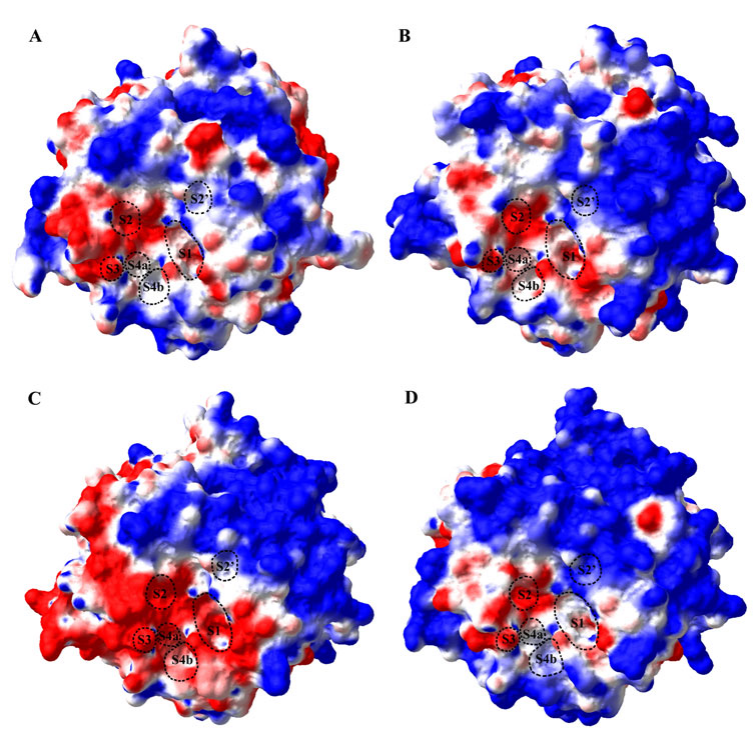
**Comparison of the electrostatic surface potentials of (A) 1IC6, (B) PR1, (C) VCP1 and (D) Ver112**. The positive surface potential is colored blue and the negative surface potential is colored red. The approximate locations of the substrate-binding sites/pockets, S2', S1, S2, S3, S4a and S4b are labeled on the surfaces.

The substrate-binding region in subtilases can be described as a surface channel or crevice capable of accommodating at least six amino acid residues (P4-P2') of a polypeptide substrate or pseudo-substrate such as an inhibitor [3]. Both the main chain and side chain interactions between the enzyme and the substrate/inhibitor contribute to binding [3,4]. In the crystal complex between proteinase K and methoxysuccinyl-Ala-Ala-Pro-Ala-chloromethyl ketone [PDB:3PRK], the P4-P1 residues of the substrate slot in between the extended enzyme backbone segments 100–104 and 132–136 to form the central strand of a three-stranded antiparallel β sheet [26]. The leaving portion P1'–P2' of the substrate runs along the enzyme backbone segment 220–222 and appears to be less tightly bound. Based on the multiple sequence alignment and crystal structures of 3PRK and 1IC6, the substrate-binding sites of the cuticle-degrading enzymes are characterized (Figure 1, 2 and 5) and briefly described as follows.

The S2' site is a hydrophobic pocket formed primarily by residues 192, 221 and 222. The S1 is a distinct, large and elongated pocket that is primarily formed at the side and bottom by segments 132–135 and 158–161, respectively. In addition, the S1 pocket is also surrounded at the rim by residue 162, at the bottom end by 169, and at the top by segment 222-225 carrying the nucleophilic residue Ser224, respectively. The S2 is a less distinct and smaller pocket than S1. It is bounded at one side by residue 100 and at the other side by the active site residue His69, at the bottom by both the hydrophobic residue 96 and the active site residue Asp39, at the bottom end by residue 40, and at the rim by residue 67, respectively. S3 is not a pocket because the side chain of the residue (Ser101 in all four enzymes) at the S3 site points away from the substrate-binding region/surface, leading to the formation of a relatively protrudent patch on the protein surface (Figure 5). The other residue that may also get involved in the interaction with the P3 side chain is at a close position 100. S4 is a very distinct pocket that is situated between two segments, 100–104 and 132–136. This pocket appears to have two subsites, S4a and S4b. S4a has residues 96 and 107 at the bottom, 133 at the side, and 102 at the rim, respectively. S4b has residues 104 at the side, 141 at the bottom, and 135 and 136 at the rim, respectively. The side chains of these residues determine the size of the S4 pocket.

Among the four investigated enzymes, the amino acid sequences in the substrate-binding sites of S2' and S3 are absolutely conserved but of S1, S2 and S4 are relatively conserved. Residue changes in the S1 pocket are found at positions 162 and 169 that are located at the rim and bottom end of this pocket, respectively. The neutral Asn162 in 1IC6 is replaced by the acidic Asp in the three fungal cuticle-degrading enzymes PR1, VCP1, and Ver112. Although Asn162 and Asp162 are practically identical in conformation, the acidic residue Asp162 would contribute to the more anionic characteristics of the S1 site in the cuticle-degrading enzymes (Figure 5), which in turn may shift the specificity to the basic P1 residues [3,4]. In addition, it has been observed that the anionic character can increase flexibility of an enzyme [27], and in particular increase flexibility in the active site region [28]. We therefore suggest that Asp162 in the cuticle-degrading enzymes would increase the flexibility of the S1 site, which in turn may enhance the catalytic efficiency of the cuticle-degrading enzymes and infection virulence by the fungi. Position 169 is occupied by the Tyr in 1IC6 and VCP1 but the Thr in PR1 and Ver112. Mutational studies on subtilisin BPN' have shown that P1 specificity can be dramatically modulated by substitution of residue at this position [29,30]. Residue changes in the S2 site are only found at position 67 with replacement of the Asn in 1IC6 by the His in PR1, VCP1 and Ver112, implying a preference of the cuticle-degrading enzymes for the negatively charged P2 residues at a low pH. The most obvious differences observed within the substrate-binding sites are located in the S4 pocket at positions 103, 104, 107, 136, and 141, in which all four enzymes contain different amino acids. For example, the Ser103 in the three cuticle-degrading enzymes in place of the Gln103 in 1IC6 would enlarge the size of the S4 pocket due to its relatively smaller side chain than that of the Gln. The Tyr104 in PR1, VCP1 and 1IC6 is observed to be replaced by the Leu in Ver112. Since previous studies have shown that the Tyr104 forms a flexible lid to the S4 pocket [3,4] of subtilases, we speculate that the Leu104 in Ver112 would broaden the entrance for P4 residues and simultaneously increase the hydrophobic property of the S4 pocket. The Ile107 in 1IC6, PR1 and Ver112 is replaced by the Val in VCP1 and the Val141 in 1IC6, PR1 and VCP1 is replaced by the Leu in Ver112. These two substitutions likely have a minor influence on the size and hydrophobicity of the S4 pocket as Ile/Val107 and Val/Leu141 are largely buried at the S4 pocket bottom and are all hydrophobic residues. The substitution of Pro136 in VCP1 for Gly136 in 1IC6, PR1 and Ver112 results in a larger rim of the S4 pocket in VCP1 than in the other enzymes. In addition, the Pro136 is located at the other side of the Tyr104 with which a gate is formed at the entrance of the S4 pocket, as thus we speculate that the Pro136 substitution may influence the entry of the P4 residues with large side chains. The hydropathy score reveals that the hydrophobic values of the S4 pockets in 1IC6, PR1, VCP1 and Ver112 are 26.0, 28.6, 27.1 and 33.1, respectively, indicating that the S4 pockets in cuticle-degrading enzymes are more hydrophobic than in 1IC6.

A notable difference in the surfaces of the proteins compared here is their different electrostatic surface potentials (Figure 5). Large parts of the surface of PR1 and Ver112 are positively charged, reflecting that these two proteases contain more positively charged residues than 1IC6 and VCP1 (Table 1). Although 1IC6 and VCP1 contain the same number of positive/negative residues (Table 1), the negatively charged surfaces are relatively evenly dispersed on the positively charged or less charged surfaces in 1IC6 (Figure 5A), whereas in VCP1 (Figure 5C), the negatively charged surfaces are mainly concentrated on regions that are part of or in close vicinity to the substrate-binding sites, and the positively charged surfaces are concentrated on the other regions.

## Discussion

The 3D structural models of the three cuticle-degrading enzymes, PR1, VCP1 and Ver112, have been modeled using the homology modeling technique. According to Baker and Sali [31], high-accuracy homology models with about 1.0 Å RMS error for the main-chain atoms can be obtained given more than 50% sequence identity to their templates, and the calculated models are comparable to the accuracy of a medium-resolution NMR or X-ray structure. Since the sequence identities of the three cuticle-degrading enzymes to the template 1IC6 are all greater than 60% and there is no large insertion or deletion relative to 1IC6, the modeled structures should be fairly accurate and can be used for predicting the Ca^2+ ^binding site, disulfide bridge, hydrogen bond, salt bridge, substrate specificity and catalytic activity. Model assessments using the program PROCHECK [32] reveal that for structural models of PR1, VCP1, and Ver112, the residues located in the most favored regions of the Ramachandran plot are 85.8%, 86.2%, and 86.0%, and in the additionally allowed regions are 13.8%, 12.9%, and 13.6%, respectively. Only one residue, the Arg126 in Ver112 is located in the disallowed region. Interestingly, this Arg is located in a loop region (the "hinge" loop) between α3 and β4, and it can be replaced by Gly in the isoform of Ver112, resulting in an amino acid polymorphism at position 126 [20]. The amino acid polymorphisms will be discussed below.

**Figure 6:**
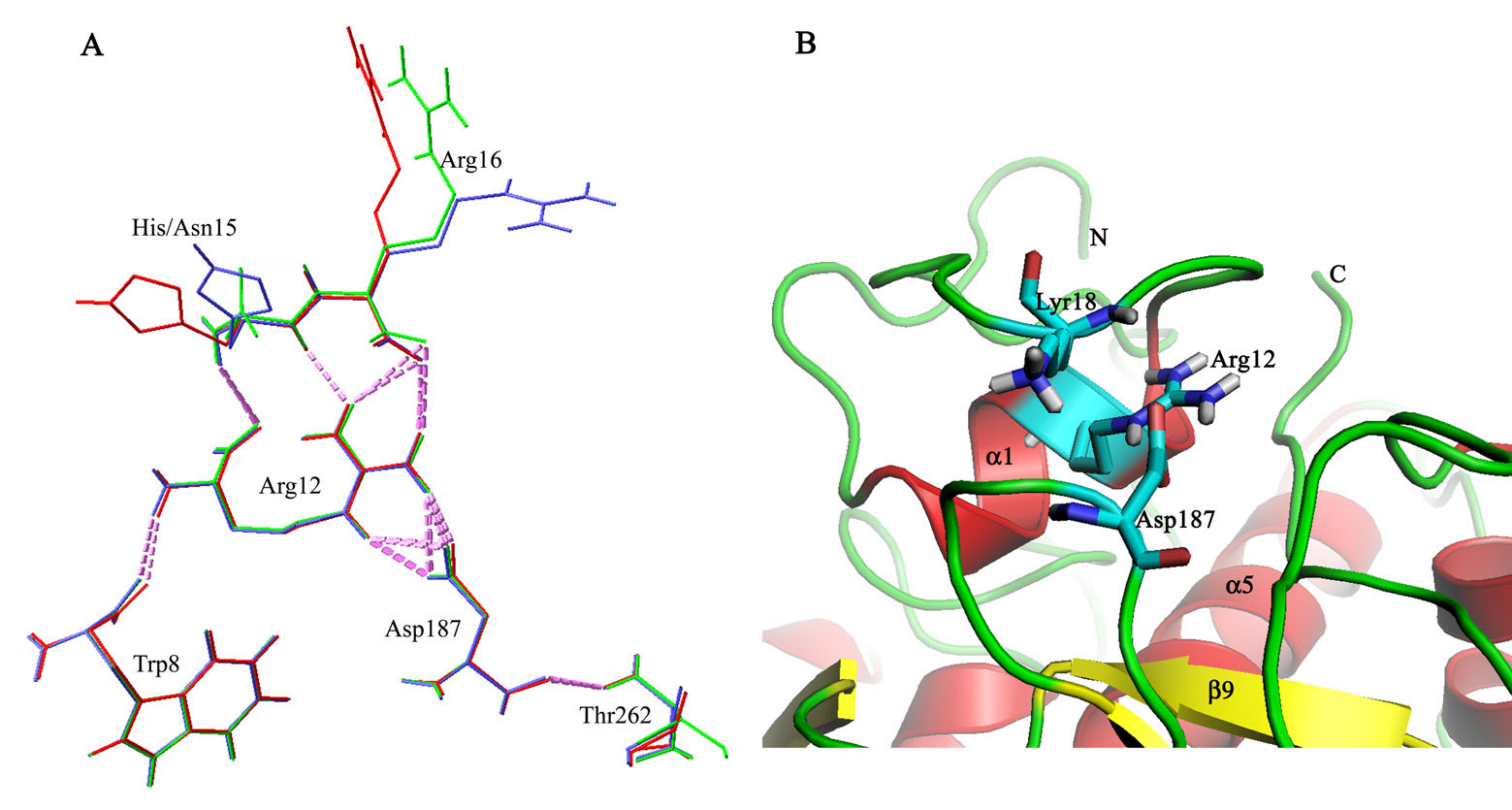
**Conserved hydrogen bond and salt bridge networks involved in stabilizing the N- and C-terminal regions**. (A) Hydrogen bond networks Arg12-[Trp8, His/Asn15, Arg16, Asp187] and Asp187-[Arg12, Thr262]. The residues from PR1, VCP1 and Ver112 are colored blue, green and red, respectively. (B) An example of the salt bridge network Arg12:Asp187:Lyr18 in PR1. This salt bridge network also occurs in VCP1 and Ver112.

Our analyses have shown that the three cuticle-degrading enzymes have not only similar physiochemical properties and optimal reaction conditions, but also highly comparable structural models with limited conformational differences observed in only some surface loops and the N-, C-termini. Furthermore, they also show comparable geometrical properties and certain conserved structural features such as the calcium binding site, disulfide bridge, salt bridge and aromatic interaction which can contribute to the overall stability of the enzymes. In terms of calcium binding site, the three cuticle-degrading enzymes contain no Ca2 that has been shown to stabilize the N- and C-terminal regions in 1IC6. However, several hydrogen bonds and salt bridge networks observed within or close to the N- and C-terminal regions of the cuticle-degrading enzymes would be a substitution for the stabilizing effect by Ca2 in 1IC6. For example, two conserved hydrogen bond networks, Arg12-[Trp8, His/Asn15, Arg16, Asp187] and Asp187-[Arg12, Thr262], and one conserved salt bridge network, Arg12:Asp187:Lyr/Arg18, are found in the cuticle-degrading enzymes to participate in stabilizing the N-, C-termini (Figure 6). These results may explain why the presence of EDTA has a minor effect on the activity of these proteinase K-like proteases [9,33]. Interestingly, the abundant hydrogen bonds and salt bridges involved in stabilizing PSL of the cuticle-degrading enzymes may play an equivalent role as another calcium binding site found in 1SH7 [24] and 1THM [25].

Although the total quantities of hydrogen bonds and salt bridges are comparable among the three cuticle-degrading enzymes, the differences in their number, type, and distribution in regions that are part of or in close vicinity to the substrate-binding sites can have a significant effect on the local flexibility, which will influence the substrate binding, catalytic activity and substrate specificity of these enzymes. Helland et al. [34] has argued that a strong hydrogen bond network, Asn97-[S99, S101], is able to bring the S2-loop of a proteinase K-like enzyme, SPRK from the psychrotroph *Serratia *species [PDB:2B6N], into a 'hub and spokes' arrangement, which confers more rigidity to the S2-loop and reduces the binding affinity (higher K_M _value) of SPRK towards the synthetic substrate suc-Ala-Ala-Pro-Phe-nitroanilide. In addition, the tight S2-loop is also able to influence the substrate specificity profile of SPRK [34]. With regard to the three cuticle-degrading enzymes compared here, a conserved hydrogen bond network, Asp97-[Gly100, S101], and a conserved salt bridge network, Asp65:Lyr94:Asp97, are found in the S2-loops. However, the number of hydrogen bonds involved in stabilizing the S2-loop is 4, 4 and 6 in PR1, VCP1 and Ver112, respectively, and the number of salt bridges involved in stabilizing the S2-loop is 1, 1, and 3 in PR1, VCP1, and Ver112, respectively. Therefore the S2-loop appears to be more rigid in Ver112 than in the other two enzymes, and it is not unreasonable to believe that the relatively high rigidity of the S2-loop in Ver112 would lead to the reduction in substrate affinity, which in turn would lower the catalytic efficiency for Ver112 in comparison to PR1 and VCP1.

In terms of substrate specificity, we have identified variable residues in the substrate-binding sites because any variation in specificity of subtilases is most likely to be caused by these variable residues, especially those residues whose side chains interact directly with the P1 and P4 residues of the substrate [3,4]. As previously mentioned, the substitution of Asp162 in the three cuticle-degrading enzymes for Asn in 1IC6 would not only enhance the catalytic efficiency but also shift the specificity to the basic P1 residues. The position 169 at the bottom end of the S1 pocket is occupied by either the Tyr or Thr in the four enzymes. Although Tyr is larger than Thr, the S1 pocket in 1IC6 and VCP1 remains as wide as that in PR1 and Ver112 as the side chains of these two residues rotate away from the S1 pocket bottom end (Figure 5). In addition, docking data has shown that this Tyr in 1IC6 is not likely to interact directly with the P1 residue of a substrate [34]. For such interactions to occur, the side chain will have to rotate, and hence, cause steric clashes with the enzyme main chain that will require energy to resolve. Taken together, we suggest that the residue substitution observed at position 169 may have a minor effect on substrate specificity for the three cuticle-degrading enzymes. On the contrary, the S4 site may contribute significantly to the substrate specificity because of the five observed variable residues in the S4 pocket. Briefly, the cuticle-degrading enzymes have larger and more hydrophobic S4 pockets than that of the 1IC6. The difference in size is mainly caused by the substitution of the smaller Ser in the cuticle-degrading enzymes for Gln in 1IC6 at position 103. In addition, Ver112 may have a preference for larger and more hydrophobic P4 residues than the other two cuticle-degrading enzymes due to the Leu substitution for Tyr at position 104, and VCP1 may have a preference for relatively smaller P4 residues due to the Pro substitution for Gly at position 136. Furthermore, the differences in conformational flexibility of the substrate-binding regions may also contribute to substrate specificity due to the induced fit process of the substrate binding [26]. It is therefore reasonable to believe that the more rigid S2-loop in Ver112 than in PR1 and VCP1 might cause variation in the specificity profile. It should be pointed out that, although the possible differences in substrate specificity between the three cuticle-degrading enzymes have been predicted from our models, more kinetic data with substrates which mimic the real "target" of the proteolytic attack should be obtained in order to verity these predictions.

The common electrostatic surface feature shared by the three cuticle-degrading enzymes is that their substrate-binding pockets are largely negatively charged while most of the other surfaces are positively charged. As mentioned above, the anionic feature of the substrate-binding regions would increase the local conformational flexibility of them, which in turn would enhance the catalytic efficiency. In addition, the large patches of the positively charged surfaces increase the adsorption of the cuticle-degrading enzymes to cuticles carrying abundant acidic residues [19,35], and also explain why the cuticle-degrading enzymes studied here are all alkaline proteases.

Some isoforms of PR1, VCP1 and Ver112 carrying the amino acid polymorphism sites have been cloned and sequenced. For example, up to 18 residue variations within PR1 of several isolates from different hosts have been detected [36]. In addition, the number of the amino acid polymorphisms detected for VCP1 and Ver112 is 2 and 1, respectively. However, no polymorphism has been found at the catalytic residue triad of the Asp, His and Ser, indicating a strong selective pressure for the maintenance of the structure/function of this site. Some of the polymorphisms observed near the substrate-binding regions may have effects on the substrate utilization, kinetics or surface property of the enzymes [37]. It has been suggested that the abundant polymorphisms within PR1 may render its isoforms recalcitrant to some of the antiproteolytic hemolymphal peptides and could enable the pathogenic fungus *M. anisopliae *to exploit a wider range of substrates [38]. For VCP1, its two polymorphism sites are observed at positions 62 and 99 [19]. The polymorphism at position 62 is the replacement of an acidic residue, Glu in the root knot nematode isolates by a neutral residue, Gln in the cyst nematode isolates. This substitution is far away from the substrate-binding regions and may have a negligible influence on the local conformation because the Glu is similar in size to Gln. However, this substitution reduces the negative charge of the VCP1 isoform which in turn enhances enzyme adsorption to the acidic cuticles [19]. The other polymorphism at position 99 is the replacement of a Gly in the root knot nematode isolates by an Ala in the cyst nematode isolates. This replacement is situated in the S2-loop and we speculate that the smaller Gly in VCP1 isoforms from the root knot nematode isolates would increase flexibility of the S2-loop and reduce steric hindrance of the larger P2 and P3 residues with corresponding substrate-binding sites, the S2 and S3. Indeed, substrate utilization assays [19] reveal that VCP1 isoforms from the root knot nematode isolates have a higher activity towards Suc-Ala-Val-Pro-Phe-pNA than towards Suc-Ala-Ala-Pro-Phe-pNA. As mentioned above, only one polymorphism at position 126 has been found so far in Ver112. This polymorphism is the replacement of the Arg126 in isolates *L. psalliotae *112 and 608 by the Gly in isolate *L. psalliotae *602 [20]. On the one hand, Arg126 increases the positive charge of VCP1, which in turn enhances the adsorption of the enzyme to the negatively charged nematode cuticle. On the other hand, Arg126 is located in the 'hinge' loop that is responsible for modulation of the orientation of the substrate-binding segments 100–104 and 132–136. Although the "hinge" loop is stabilized by the disulfide bridge Cys34–Cys123 and the salt bridge Asp117:Arg121, Arg126 may make it unstable due to the large size of Arg and no apparent negatively charged complementary residue being found to form the salt bridge. We speculate, therefore, that the Arg126 would make the "hinge" loop more flexible and influence the orientation of the substrate-binding segments (100–104 and 132–136) and the catalytic activity. However, more kinetic data should be obtained to clarify these effects. In addition, the host related amino acid polymorphism in PR1, VCP1 and Ver112 between different isolates may also contribute to host preference by fungi.

## Conclusion

The 3D structural models of the three cuticle-degrading proteases, PR1, VCP1 and Ver112, have been modeled using the homology modeling technique. These models reported here are very similar to each other and to the template structure of 1IC6, with restricted conformational differences found only in certain surface loops and the N-, C-termini. These structures share the common disulfide bridges and similar calcium binding sites, physiochemical properties, optimal reaction conditions and high sequence identities. These similarities support the initial hypotheses that the three cuticle-degrading enzymes belong to the proteinase K-like serine protease family. These enzymes secreted by nematophagous and entomophagous fungi can be considered to be general-purpose proteases because they are required either for degrading cuticles or for growth on purified proteinaceous cuticles. However, the variable residues observed within the substrate-binding sites and the different conformational flexibilities in the S2-loop can be expected to cause variation in substrate specificity as well as in catalytic activity. The host related amino acid polymorphisms observed for these cuticle-degrading enzymes are not only of great interest to the functional pathology of fungi, but also to future exploitation of these fungi as biocontrol agents.

## Methods

### Homology modeling

The protein sequences for PR1 [15], VCP1 [19] and Ver112 [20] were obtained from the GenBank database with accession numbers [GenBank:S22387], [GenBank:AJ427460] and [GenBank:AY692148], respectively. The signal peptide sequences and the pro-peptide sequences were removed and the obtained mature proteins consisted of 281, 281, and 280 amino acids for the PR1, VCP1 and Ver112, respectively. The X-ray structure of the serine protease proteinase K [23] ([PDB:1IC6], hereafter it is referred to as 1IC6 according to its PDB accession number) from *Tritirachium album limber *at 0.98 Å resolution was used as a template to model the 3D structural models of PR1, VCP1 and Ver112. All modelings were performed using the MODELLER 7v7 software package [39] running under the Linux operating system. Briefly, to obtain the structural model of PR1, the target sequence was first aligned with the template sequence of 1IC6 using an in-house written script "alignment" for the MODELLER 7v7. Second, 20 PR1 models were generated from the template 1IC6 using an in-house written script "get-model". The insertions and deletions were generated and optimized using a loop modeling sub-routine within the "get-model" script with a 3D_INTERPOLATION algorithm and a thorough optimization protocol (a thorough molecular dynamics/simulated annealing procedure and the MD_LEVEL is set to refine_3). Finally, these 20 models were clustered and an optimized average model was generated using a MODELLER 7v7 script "cluster". The transferred coordinates for a given target atom were the average of the 20 models in the largest cluster of the corresponding atom with the cluster cut off at 1.5 Å. The modeling procedures for VCP1 and Ver112 were identical to that for the PR1. To further optimize their structures, the four proteases (including protease K, 1IC6) were respectively subjected to the steepest descent energy minimization with the tolerance 100 kJ mol^-1 ^nm^-1^, followed by a second conjugate gradient energy minimization with the tolerance 10 kJ mol^-1 ^nm^-1 ^using the program "mdrun" within the GROMACS software package [40]. For PDB files of the homology models after energy minimizations, [seeAdditional file 1: file1_PR1.pdb], [see Additional file 2: file2_VCP1.pdb] and [see Additional file 3: file3_Ver112.pdb]. The overall geometrical qualities of these models were assessed using the program PROCHECK [32].

### Physicochemical property calculations

The molecular weight, amino acid composition and charge distribution of each mature protease were calculated using the program SAPS [41]. The isoelectric point (pI) and the extinction coefficient (EXTCOEF) at 280 nm of the mature enzymes were calculated using the pI service [42] and the EXTCOEF [43] program, respectively.

### Structural analyses and comparisons

The geometrical properties of the mature proteases such as the radius of gyration (Rg), number of native contacts (NNC), number of hydrogen bonds (NHB), and number of salt bridges (NSB) were calculated using the programs g_gyrate, g_mindist, g_hbond and g_saltbr within the GROMACS software package [40], respectively. Pairwise superposition of structures was performed with the g_confrms program within GROMACS. Secondary structure element and solvent accessible surface area (SASA) of the models were analyzed with the DSSP program [44]. The electrostatic surface potential was calculated using the Swiss-pdbViewer software package [45]. The hydrogen network was represented by residue1-[residue2, residue3, residue4...], where residue1 could form hydrogen bonds with residue2, residue3 and residue4, etc. The salt bridge network was represented by residue1:residue2:residue3, where residue2 could form salt bridges with residue1 and residue3.

### Amino acid sequence alignment

Multiple sequence alignment was performed using the CLUSTALW program [46] with the gap open and extension penalties 10.0 and 0.20, respectively. Next, the obtained multiple sequence alignment was improved by manual adjustments according to the known alignments from MODELLER 7v7 and the superimpositions of 3D structural models. The amino acid numbering used throughout this paper corresponds to that of 1IC6. Substrate-binding site residues in the S2', S1, S2, S3, S4 sites of the cuticle-degrading enzymes were assigned based on those of 1IC6 and the crystal structure of proteinase K in complex with an inhibitor Methoxysuccinyl-Ala-Ala-Pro-Ala-chloromethyl ketone [PDB:3PRK] [26]. The nomenclature of the substrate residues and the substrate-binding sites of enzymes is according to Schechter and Berger [47]. The scissile peptide bond is located between the P1 and P1' substrate residues and residues of the substrate are numbered P2', P1', P1, P2, P3, P4, etc. towards the N-terminus. The complementary sites of the substrate-binding region of the enzyme are correspondingly numbered S2', S1', S1, S2, S3, S4, etc.

## Authors' contributions

SQL and ZHM contributed equally to this work. SQL and ZHM participated in the design of the study, performed the structural modeling, carried out the structural analysis, and drafted the manuscript. JKY participated in the sequence alignment and experimental data collection. YXF and KQZ designed the study and helped to draft the manuscript. All authors read and approved the final manuscript.

## Supplementary Material

Additional file 1 PDB file of the homology model of PR1 after energy minimizations. Click here for file

Additional file 2 PDB file of the homology model of VCP1 after energy minimizations. Click here for file

Additional file 3 PDB file of the homology model of Ver112 after energy minimizations. Click here for file

## Figures and Tables

**Table 1:** Physiochemical properties and optimal reaction conditions of the proteinase K (1IC6) and the three cuticle-degrading enzymes (PR1, VCP1 and Ver112).

Protease	Fungus	Host	MW^a ^(kDa)	NAA^b^	pI^c^	RK/DE^d^	EXTCOEF^e ^(M^-1^cm^-1^)	Inhibitor	SI^h ^(%)	Optimum^i^	
										
										pH	T ^j ^(°C)
1IC6	*Tritirachium album*	NA	28.9	279	7.71	20/19	33200	PMSF^f^ DFP^g^	100	7.5–12	50–60
PR1	*Metarhizium anisopliae*	Insect	28.6	281	8.37	21/18	26800	PMSF	66.4	8–10	50–60
VCP1	*Pochonia chlamydosporia*	Nematode	28.5	281	7.74	20/19	28650	PMSF	62.5	8–10	50–60
Ver112	*Lecanicillium psalliotae*	Insect	28.6	280	8.67	22/18	27370	PMSF	64.1	8–10	50–60

^a ^Calculated molecular weight.

^b ^Number of amino acids.

^c ^Predicted isoelectric point.

^d ^Number of positively charged residues/number of negatively charged residues.

^e ^Predicted extinction coefficient at 280 nm.

^f ^Phenyl methane sulphonyl fluoride.

^g ^Diisopropyl fluorophosphates.

^h ^Sequence identity with respect to 1IC6.

^i ^Optimal reaction conditions.

^j ^Temperature.

**Table 2:** Geometrical properties of the proteinase K (1IC6) and the three cuticle-degrading enzymes (PR1, VCP1 and Ver112).

Protease	RMSD^a ^(Å)	SASA^b ^(Å^2^)	Rg^c ^(Å)	NHB^d^	NNC^e^	NSB^f^	ENE^g^	DIH^h^	SSE^i^		
									
									α helix	β sheet	Turn
1IC6	0	109.2	1.67	223	134896	14	-9288.2	0	69	66	43
PR1	0.75	113.4	1.67	189	129252	12	-8560.3	0	67	66	42
VCP1	0.75	111.0	1.67	189	128896	12	-8560.0	0	70	64	47
Ver112	0.58	106.6	1.66	195	132572	13	-8732.7	1	70	66	43

^a ^Backbone RMSD with respect to 1IC6.

^b ^Total solvent accessible surface area.

^c ^Radius of gyration.

^d ^Number of hydrogen bonds. A hydrogen bond is considered to exist when the donor-hydrogen-acceptor angle is larger than 120° and the donor-acceptor distance is smaller than 3.5 Å.

^e ^Number of native contacts. A native contact is considered to exist if the distance between two atoms is less than 6 Å.

^f ^Number of salt bridges. A salt bridge is considered to exist if the distance between two oppositely charged residues is within 6 Å.

^g ^Potential energy after energy minimization in the GROMOS96 force field.

^h ^Number of residues in the disallowed regions in the Ramachandran plot.

^i ^Number of residues in the corresponding secondary structure elements.
